# Removing Phosphorus from Aqueous Solutions Using Lanthanum Modified Pine Needles

**DOI:** 10.1371/journal.pone.0142700

**Published:** 2015-12-02

**Authors:** Xianze Wang, Zhongmou Liu, Jiancong Liu, Mingxin Huo, Hongliang Huo, Wu Yang

**Affiliations:** 1 School of Environment, Northeast Normal University, Changchun, China; 2 Jilin Engineering Research Centre for Municipal Wastewater Treatment and Water Quality Protection, Changchun, China; University Medical Center Utrecht, NETHERLANDS

## Abstract

The renewable pine needles was used as an adsorbent to remove phosphorus from aqueous solutions. Using batch experiments, pine needles pretreated with alkali-isopropanol (AI) failed to effectively remove phosphorus, while pine needles modified with lanthanum hydroxide (LH) showed relatively high removal efficiency. LH pine needles were effective at a wide pH ranges, with the highest removal efficiency reaching approximately 85% at a pH of 3. The removal efficiency was kept above 65% using 10 mg/L phosphorus solutions at desired pH values. There was no apparent significant competitive behavior between co-existing anions of sulfate, nitrate, and chloride (SO_4_^2-^, NO_3_^-^ and Cl^-^); however, CO_3_^2-^ exhibited increased interfering behavior as concentrations increased. An intraparticle diffusion model showed that the adsorption process occurred in three phases, suggesting that a boundary layer adsorption phenomena slightly affected the adsorption process, and that intraparticle diffusion was dominant. The adsorption process was thermodynamically unfavorable and non-spontaneous; temperature increases improved phosphorus removal. Total organic carbon (TOC) assays indicated that chemical modification reduced the release of soluble organic compounds from 135.6 mg/L to 7.76 mg/L. This new information about adsorption performances provides valuable information, and can inform future technological applications designed to remove phosphorus from aqueous solutions.

## Introduction

Phosphorus plays a critical role in the development of ecosystems, agriculture, and industry, but also becomes a pollutant in water bodies [1, 2]. To meet increasing demands for phosphorus, more and more phosphate ore reserves are being exploited. However, current reserves will be depleted in 50–100 years [3–5], highlighting the important need to identify new ways to recycle and reuse phosphorus [6].

Biosorbents are important tools for chemical recover and recycling. They are environmentally friendly, cost-effective, and easily available from industrial, agricultural, and other types of biomasses [7, 8]. Biosorbents contain a large number of hydroxyl group enriched components, such as cellulose, hemicellulose, and lignin; these easily facilitate a wide range of chemical reactions [9]. Compared to other methods [10, 11], chemical modifications can effectively change biomass sorbent properties and enhance removal efficiencies. These modifications also control the release of soluble organic compounds to reduce the total organic carbon (TOC). Biosorbents have been used to treat wastewater, and have a demonstrated ability to purify wastewater with low phosphorous concentrations [10].

Modified media including ammonium-type chemicals and metal hydroxides (oxides). Modifying ammonium chemicals (e.g. animated intermediate [12] and urea [13]) could improve content of nitrogen and phosphorus, enhancing the feasibility of using biosorbents as fertilizers. Past studies have reported that biosorbents modified with transitional elements, such as Fe [10, 14–16], La [16, 17] and Zn [18], could serve as materials for efficient phosphorus desorption. Using metal-loaded biosorbents and applying to soil using strict guidelines [19], could allow metals on biosorbent surfaces to improve soil microbial activity, crop quality, and plant growth [20]. Meanwhile, biomass sorbents themselves are biodegradable natural cellulose [21] and contain macronutrients (N, P). As such, they may improve the soils’ chemical, physical and biological properties [22]. This possibility is supported by the fact that farmers in Europe and North America are increasingly ploughing straw and other biomass into agricultural soils [23]. Using biomass sorbents to recycle phosphorus could reframe the perception that phosphorus is a contaminant to instead positioning it as a resource. This would provide a new approach for phosphorous recovery and reuse, from both organisms and wastewater.

Lanthanum hydroxides (oxides) are environmentally friendly materials [24] that have been proven to have high phosphorus adsorption capacities and high removal efficiencies [25–28]. However, these materials also have drawbacks, such as difficulty in separating small particles [24–28], restrictions in effective pH [16, 17], complex synthesis and materials processing [25], and outflow easily [26] blocking practical applications. Pine needles are a kind of renewable resource that regenerate quickly; pine trees are widely present in northern China, leading to a high accumulation of pine needles. Pine needles are mainly composed of cellulose, hemicellulose, lignin, and other low molecular weight compounds [29, 30] and can be easily modified by metal hydroxides (oxides). In this study, LH was loaded on the surface of AI pine needles to solve the problems of adsorbent particles separation and aggregates. The study evaluated the adsorption isotherms, kinetics, effect of pH, effect of co-existing anions, and soluble organic compound release.

## Materials and Methods

### 2.1 Chemicals

All chemicals used for this study were analytical grade, purchased from Tianjin Fuchen Chemical; all solutions were prepared with distilled water. Lanthanum nitrate hexhydrate (La(NO_3_)_3_·6H_2_O) was used to prepare the adsorbent LH pine needles. Phosphorus stock solutions were prepared using potassium dihydrogen phosphorus (KH_2_PO_4_).

### 2. 2 Preparation of LH pine needles

**Fig 1:**
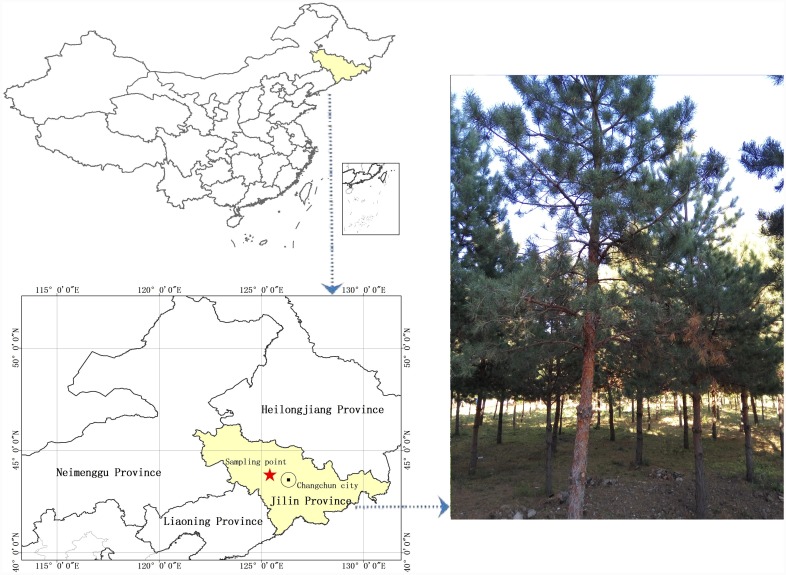
Location of the pine needles collection.

Pine needles were gathered from the woods (shown in Fig 1, we got full permission from Northeast Normal University for collecting the pine needles from the woods that belongs to NENU, and the geographic coordinates is 125.43° E, 43.83° N) and washed 5 times with distilled water to remove dust. The samples were then dried overnight at a temperature of 353 K. The needle samples were then crushed to a size of approximately 1–2.5 mm in length, and then soaked in a mixture of 3M sodium hydroxide (NaOH) and isopropanol (V _NaOH_/V _isopropanol_ = 1:1) for 24 hours. The soaked pine needles were then washed again with distilled water and dried overnight, again at a temperature of 353 K. The cooled AI pine needles were then set aside for further use.

LH pine needles were synthesized using the following procedures: 20 g of AI pine needles were added to 250 mL 0.1 M La(NO_3_)_3_, stirred for 1 h, and deposited for 5 h. An ammonia solution (28%, wt) was then added drop by drop to adjust the pH to 8–9. The samples were again deposited for 10 h, washed 5 times with distilled water, and dried at 353 K for 720 min.

### 2.3 Batch adsorption experiments

Batch experiments were conducted to investigate the phosphorus adsorption performances of AI pine needles and LH pine needles. Samples were placed into multiple 150 mL glass stoppered conical flasks containing 50 mL phosphorus solutions at target concentrations. Flasks were then placed on a rotary shaker and stirred at 160 rpm at a temperature of 298 K. Samples of 0.1 g AI pine needles were added to a series of solutions with different phosphorus concentrations (0, 1, 2, 5, 10, 15, 20, 30, 40 and 50 mg/L) for 720 min at temperatures of 288, 298, and 308 K. This was done to evaluate the effect of initial phosphorus concentrations and to support isotherm studies.

For kinetics studies, 1 g of LH pine needles was added to 500 mL of 5 mg/L phosphorus solution for 1,440 min. Samples of 2 mL were then collected at different time intervals (0, 2, 5, 10, 20, 30, 45, 60, 90, 120, 180, 240, 360, 480, 600, 720, 1440 min). The effect of initial pH was studied by mixing 0.1 g LH pine needles with 10 mg/L phosphorus solution at pH values ranging from 3 to 10 for 720 min. Solution pH was adjusted with 0.1 mol/L HCl and NaOH. To start the effect of coexisting anions, 0.1 g LH pine needles were added into 5 mg/L phosphrous solutions, which contained 1, 5, 10, 50 or 100 mg/L of SO_4_^2-^, CO_3_^2-^, NO_3_^-^, or Cl^-^ ions, individually. The release of soluble organic compounds (determined by TOC) was conducted by mixing 0.1 g pine needles, AI or LH pine needles with 5 mg/L phosphorus solution for 720 min.

All the experiments were carried out in triplicate.

### 2.4 Analytical methods

Surface morphologies and TOC analyses were obtained using a PHILIPS XL30 ESEM with an SUTW-Sapphire system and TOC-L CPH (Shimadzu, Japan), respectively. Phosphorus concentrations were determined using a Metrohm 881 ion chromatograph coupled with a Metrosep A Supp4 column. A solution with 1.8 mM of Na_2_CO_3_ and 1.7 mM of NaHCO_3_ was used as a mobile phase, at a flow rate of 1.2 mL/min.

The concentration of residual lanthanum ions in the solutions after the adsorption process was determined. The residual solution was collected into the syringe and filtered with 0.45 μm filter disk into a proper sample tube. Concentration of lanthanum in the filtered solutions was analyzed by an inductively coupled plasma atomic emission spectrometer (ICP) (LEEMAN Prodigy, USA).

Phosphorus adsorption capacities (*q*_*e*_, mg P/g) and removal efficiencies (%) were determined using the following equations:

$$q_{e}=\frac{V\times \left(C_{0}-C_{e}\right)}{1000\times m}$$ (1)

$$\text{Re }moval\;efficiency\;\;\;\;(\%)=(1-\frac{C_{e}}{C_{0}})\times 100\%$$ (2)

In these equations, *q*_*e*_ (mg P/g) was the phosphorus adsorption capacity, *V* (mL) was the volume of the solution, *C*_*o*_ and *C*_*e*_ (mg/L) were the concentrations before and after adsoption, and *m* (g) was the adsorbent mass.

## Results and Discussion

### 3.1 Characteristics of AI and LH pine needles

**Fig 2:**
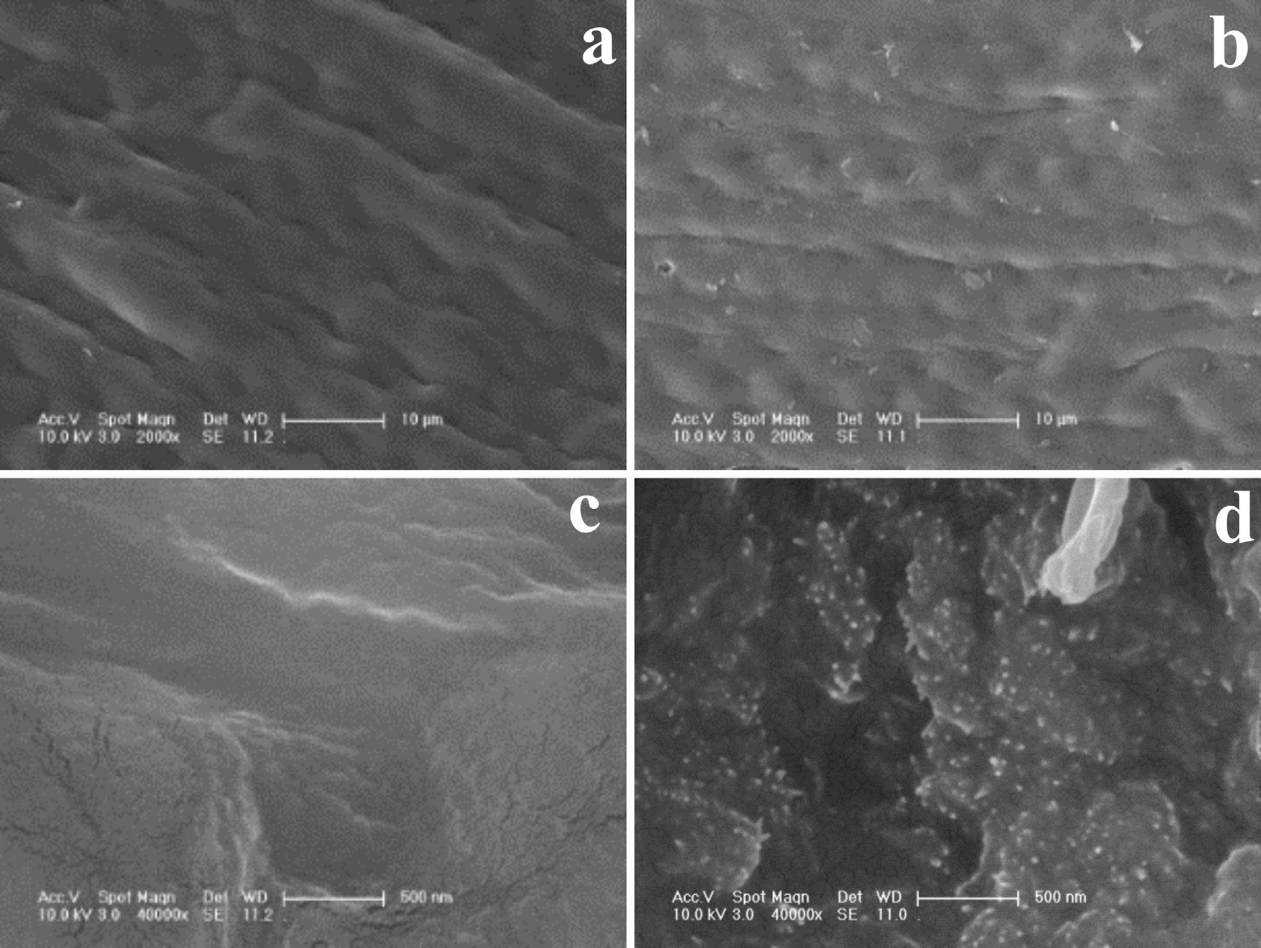
SEM images of AI pine needles and LH pine needles. (a) Outer surface of AI pine needles, (b) Outer surface of LH pine needles, (c) Interior of AI pine needles, (d) Interior of LH pine needles.

Fig 1 displays the SEM images of AI and LH pine needles. The outer surfaces of both the AI and LH pine needles had many folds, but look smooth, without significant changes (Fig 2a and 2b). This implies that the AI treatment did not undermine the needle’s outer surface lignified cuticle. However, significant changes were seen on the interior (Fig 2c and 2d). White fine spots were observed on the interior of LH pine needlesand these spots were confirmed as lanthanum oxide according to EDAX spectrum, and 11.84% (wt%) of La was apparent in LH pine needles, (S1 Fig, seen in the supporting information). The AI treatment undermined the hydrogen bonds between lignin and carbohydrates or cellulose molecules made the structure fluffy and porous [31] through the extraction of the pine needle tissue organic matter. Fibril exposure contributed to a larger specific surface area, making lanthanum attachment easier, and providing a larger accessible surface area and more reactive functional groups [32].

### 3.2 Effect of initial phosphorus concentrations

**Fig 3:**
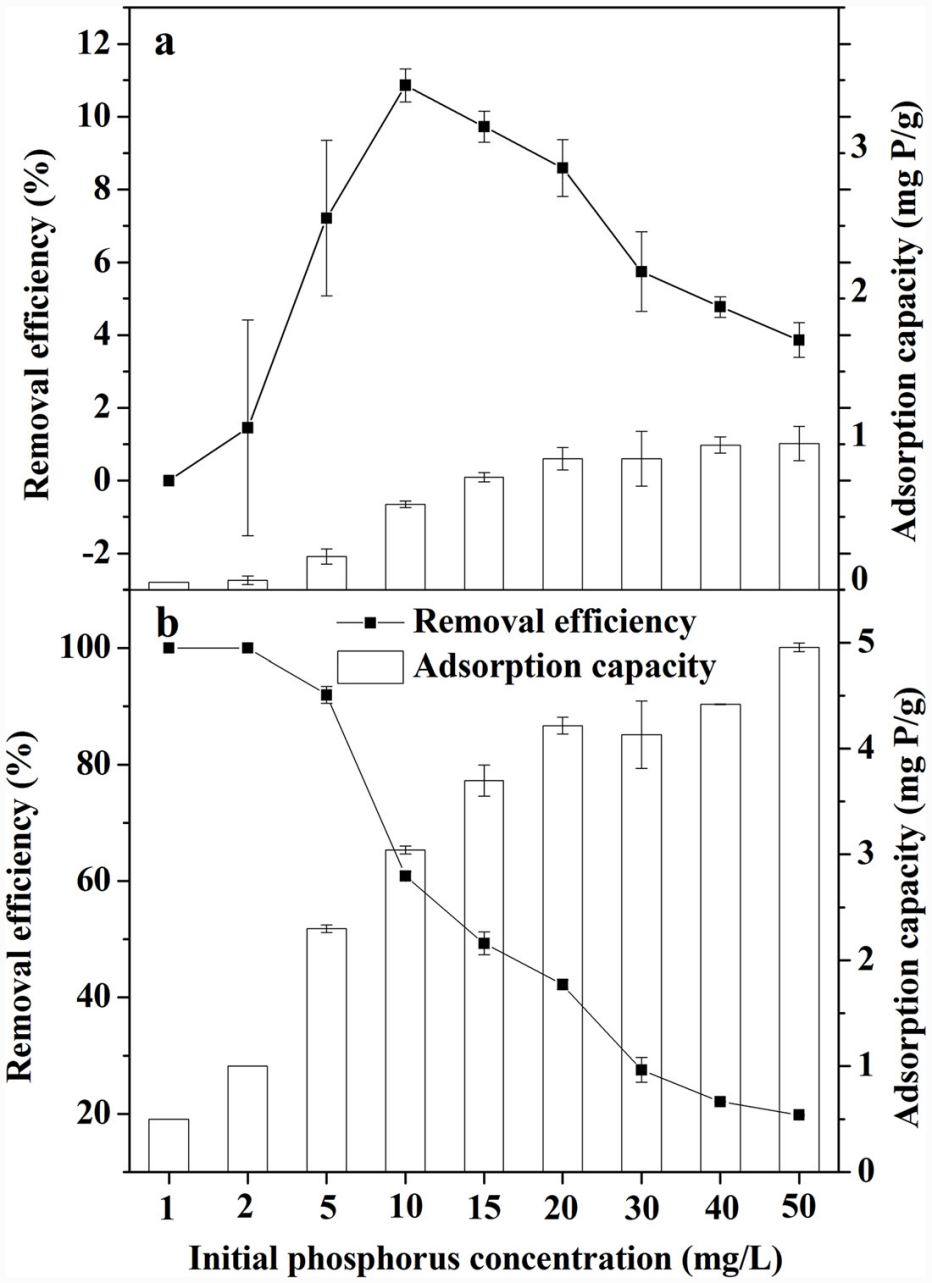
Effect of initial phosphorus concentrations, (a) AI pine needles and (b) LH pine needles.

Fig 3a shows the removal efficiency of phosphorus by AI and LH pine needles at different initial concentrations. AI pine needles were ineffective at decontaminating phosphorus from aqueous solutions; less than 10.9% of the phosphorus was removed at an initial concentration ranging from 0–50 mg/L. However, LH pine needles showed higher removal efficiency than AI pine needles under the same initial concentrations. The highest removal efficiency of 92% was obtained when the initial phosphorus concentration was 5 mg/L. Removal efficiencies decreased with increasing initial concentration since the quantities of the binding sites for a certain dose of LH pine needles were constant, and only 19.8% of the phosphorus was removed when the initial concentration increased to 50 mg/L. As Fig 3b shows, both the adsorption capacity of AI and LH pine needles were highly dependent on initial phosphorus concentrations and increased as concentrations increased. We assume that as the initial phosphorus concentrations increase, it becomes easier to overcome resistance to phosphorus mass transfers between the aqueous phase and the adsorbent surface [33].

### 3.3 Kinetics

**Fig 4:**
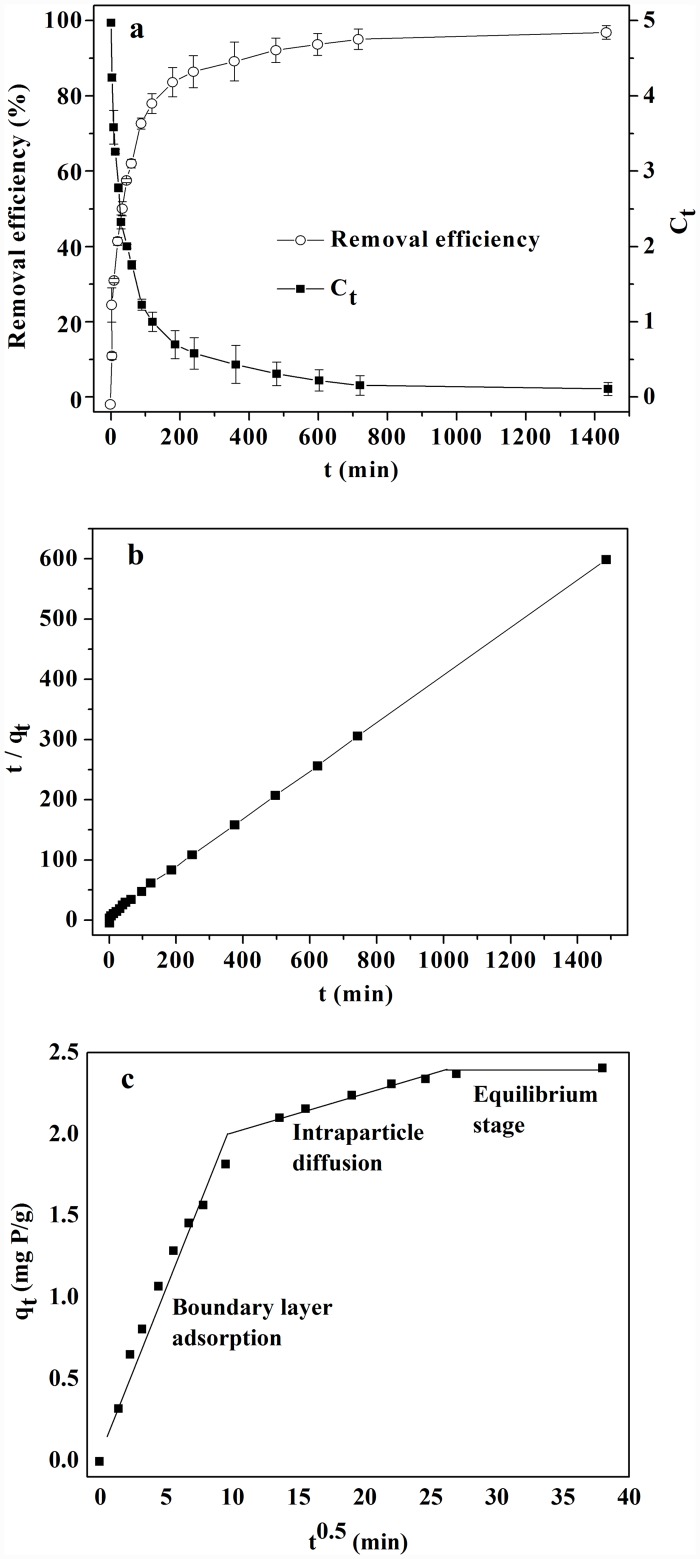
Kinetics of phosphorus removal by LH pine needles, (a) Effect of contact time on removal efficiency, (b) Pseudo second order kinetic model and (c) Intra-particle diffusion model.

The kinetics of phosphorus removal by LH pine needles were assessed using the following models [34, 35]; Fig 4 and Table 1 show the results.

**Table 1:** Kinetic parameters for phosphorus adsorption onto LH pine needles.

Pseudo second-order kinetic			
q_e (exp)_ (mg P/g)	q_e (cal)_ (mg P/g)	k_2_ (g·mg^-1^·min^-1^)	r
2.33	2.43	1.71×10^−2^	0.999
Intra-particle diffusion kinetic			
K_int_(mg·g^-1^·min^-0.5^)	Boundary layer adsorption	1.77×10^−1^	0.964
	Intraparticle diffusion	2.43×10^−2^	0.931
	Equilibrium phase	3.42×10^−3^

$$\text{ln}(q_{e}-q_{t})=\text{ln}q_{e}-k_{1}t$$ (3)

$$t/q_{t}=1/k_{2}q_{e}^{2}+t/q_{e}$$ (4)

$$q_{t}=K_{\text{int}}t^{0.5}+C$$ (5)

In these equations, *q*_*t*_ (mg P/g) is the phosphorus adsorption capacities at time *t* (min); *k*_*1*_ (min^-1^) is the adsorption rate constant of the pseudo first-order kinetic model; *k*_*2*_ (g·mg^-1^·min^-1^) is the adsorption rate constant of the pseudo second-order kinetic model; *K*_int_ (mg·g^-1^·min^-0.5^) is the rate constant of intra-particle diffusion model; *C* is the diffusion constant.

As Fig 4a shows, the removal kinetics were described as an adsorption process with three phases (rapid, low and equilibrium phases); this is consistent with previous studies [10, 16]. Nearly 85% of the total phosphorus content was removed in the rapid phase, which occurred in the first 180 min; only 11% was removed in the next 540 min. A dynamic equilibrium phase occurred between 720 and 1440 min; as such, 720 min was selected as the optimal time in the subsequent experiments. The sorption process is a better fit to the pseudo second-order model shown in the Fig 4b; the experimental values of *q*_*e(exp)*_ (2.33 mg P/g) were very close to the calculated value *q*_*e(cal)*_ (2.43 mg P/g). This suggests that phosphorus adsorption onto LH pine needles was done through chemisorption, or there was chemical bonding between adsorbent active sites [34].

The adsorbate species may be transported from the bulk solution onto the sorbents through intra-particle diffusion/transport processes [36]; as such, the rate limited step was taken into consideration and the intra-particle diffusion model was introduced. The R-values listed in Table 1 were close to unity, indicating the feasibility of the model’s application and revealing the presence of intra-particle diffusion process [36]. Based on the diffusion rate constant, the adsorption process could be divided into three phases (Fig 4c): the boundary layer adsorption phase, the intraparticle diffusion phase, and the equilibrium adsorption phase. The boundary layer adsorption phase was rapid; the intraparticle diffusion phase reflected phosphorus adsorption onto the surface of LH-treatment pine needles. Readily accessible sites reacted rapidly with phosphorus through mass transferring, followed by the same reaction occurring at progressively less accessible sites controlled by diffusion [35]. This suggests that intraparticle diffusion may govern the reaction adsortpion kinetics. Meanwhile, first phase’s straight fitting line did not pass through the origin (*C* = 0.175), indicating that the boundary layer adsorption had some effect on the adsorption process [36, 37].

### 3.4 Effect of temperature and thermodynamics analysis

**Fig 5:**
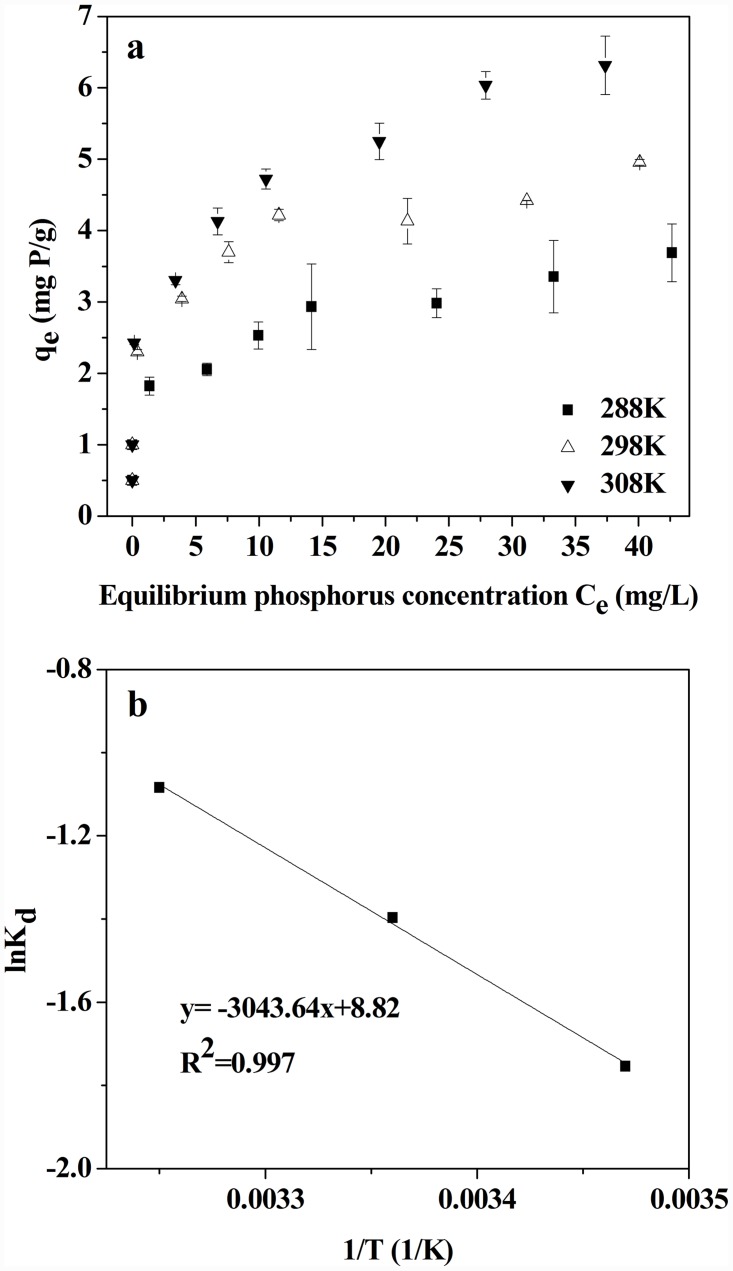
(a) Effect of temperature on phosphorus removal by LH pine needles, (b) Van’t Hoff plot of phosphorus removal by LH pine needles.

Fig 5a shows the effects of temperature on phosphorus removal by LH pine needles. The phosphorus adsorption capacity increased with increasing temperature; this is explained by the increasing penetration of ionic phosphorus into micropores of LH pine needles or the creation of new active sites at higher temperatures [38]. The results also indicate that phosphorus adsorption is an endothermic process.

According to Al-Degs’ study [38], thermodynamic parameters such as ΔG°, ΔH°, ΔS° calculated using the following equations, are important parameters to evaluate the thermodynamic feasibility and nature of the adsorption process [39].

$$\Delta G^{0}=-RT\text{ln}K_{d}$$ (6)

$$\text{ln}K_{d}=\frac{\Delta S^{0}}{R}-\frac{\Delta H^{0}}{RT}$$ (7)

$$K_{d}=\frac{aq_{e}}{C_{e}}$$ (8)

$$\Delta G^{0}=\Delta H^{0}-T\Delta S^{0}$$ (9)

In these equations, *ΔG°*, *ΔH°*, and *ΔS°* are the free energy of sorption (kJ/mol), the standard enthalpy change (kJ/mol), and the standard entropy change (J/(mol·K)), respectively; *T* (K) is the absolute temperature in Kelvin; *R* (8.314 J/(mol·K)) was the universal gas constant; *K*_*d*_ is the thermodynamic equilibrium constant; α is the adsorbent dose (g·L^-1^); *ΔH°* and *ΔS°* identify the slope and intercept of the linear plot of ln*K*_*d*_ *vs*. *1/T*, respectively.

Fig 5b and Table 2 present the thermodynamic analysis of phosphorus adsorption onto LH pine needles. The positive value of the enthalpy change demonstrates the endothermic nature of the reaction, which was supported by increases in *q*_*m*_ as temperature increases. The *ΔG°* values decreased with the increasing reaction temperature, a result of the positive entropic contribution (*ΔG° = ΔH°—TΔS°*). Unexpectedly, positive values of *ΔG°* resulted. These results are different from previous research examining phosphorus sorption to lanthanum (hydr)oxides modified materials [26, 32], it might be that the overall entropy variation decreases when the equilibrium state is attained, as species are adsorbed onto a surface [40]. This suggests the adsorption process was thermodynamically unfavorable, and a higher energy input or improving temperature were needed to ease the process.

**Table 2:** Thermodynamic parameters for phosphorus adsorption onto LH pine needles.

T(K)	K_d_	ΔG°(KJ/mol)	ΔH°(KJ/mol)	ΔS°(J/mol·K)
288	0.173	4.20		
298	0.247	3.46	25.30	73.28
308	0.338	2.78		

### 3.5 Isotherms

**Fig 6:**
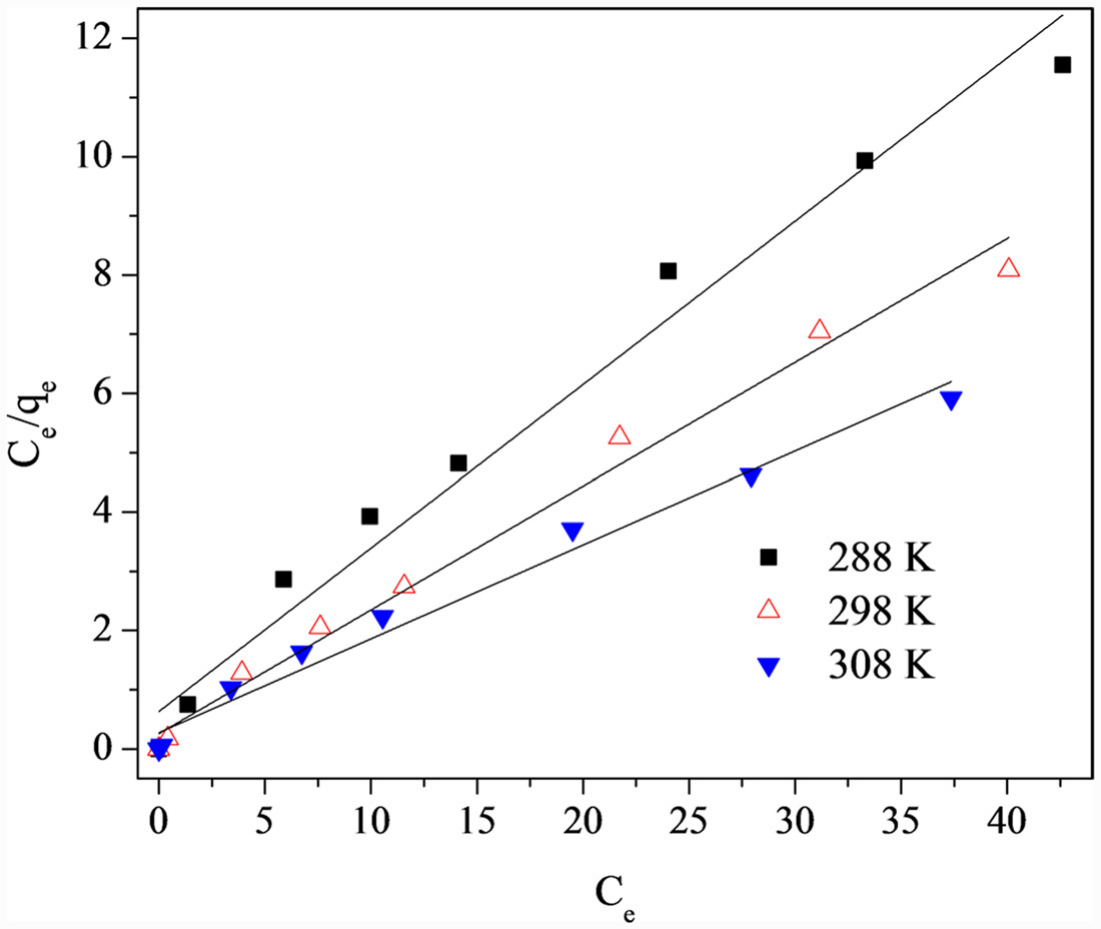
Langmuir isotherms for phosphorus removal by LH pine needles at different temperatures.

The adsorption isotherms were also calculated using Langmuir models; Fig 6 and Table 3 show the results and parameters, respectively.

**Table 3:** Langmuir isotherm parameters for phosphorus adsorption onto LH pine needles.

T (K)	*q*_*m*_ (mg P/g)	*b* (L/ mg)	*r*
288	3.64	0.437	0.977
298	4.80	0.808	0.988
308	6.31	0.576	0.981

$$q_{e}=bq_{m}C_{e}/(1+bC_{e})$$ (10)

In this equation, *q*_*m*_ (mg P/g) was the theoretical maximum phosphorus adsorption capacity; *b* (L/mg) was the affinity constant.

The Langmuir model fit the data very well (r > 0.97) at the three different temperatures. In the case of the LH pine needles, hydroxyl groups (originating from Lanthanum hydroxide and phenolic-OH or carboxylic groups of the LH pine needles) may react with phosphorus oxygen (P-O) to form monolayer adsorption [35, 41]. At 298 K, the maximum phosphorus adsorption capacities for AI and LH pine needles were 0.98 and 4.80 mg P/g, respectively. The *q*_*m*_ values may not provide an accurate estimate of the long term sorption capacity, but could still be useful for comparing alternative materials. Compared with the other biomass sorbents listed in Table 4, the adsorption capacity of modified giant reed, Fe(III) modified eggshell, and modified giant reed were all higher than that of LH pine needles; the smaller size (0.05–0.35 mm) provided a large surface to enhance the adsorption. LH pine needles have a lower adsorption capacity and no significant advantages; however, they are easily acquired and abundantly available. As such, a simple synthesis process, using LH pine needles as a biosorbent to remove phosphorous could be a feasible alternative.

**Table 4:** Phosphorus adsorption capacities of different biomass sorbents.

Adsorbents	Adsorption capacity (mg P/g)
AI pine needles	0.98
LH pine needles	4.80
Juniper fiber treated with acid mine drainage [42]	2.31
Fe(III) loaded ‘okara’ [10]	4.78
Modified aspen wood fiber [43]	4.30
ZnCl_2_ activated coir pith [18]	5.1
Metal-loaded orange waste gel [16]	13.94
Fe(III) modified eggshell [14]	14.49
Modified giant reed [44]	19.89

### 3.6 Effect of pH

**Fig 7:**
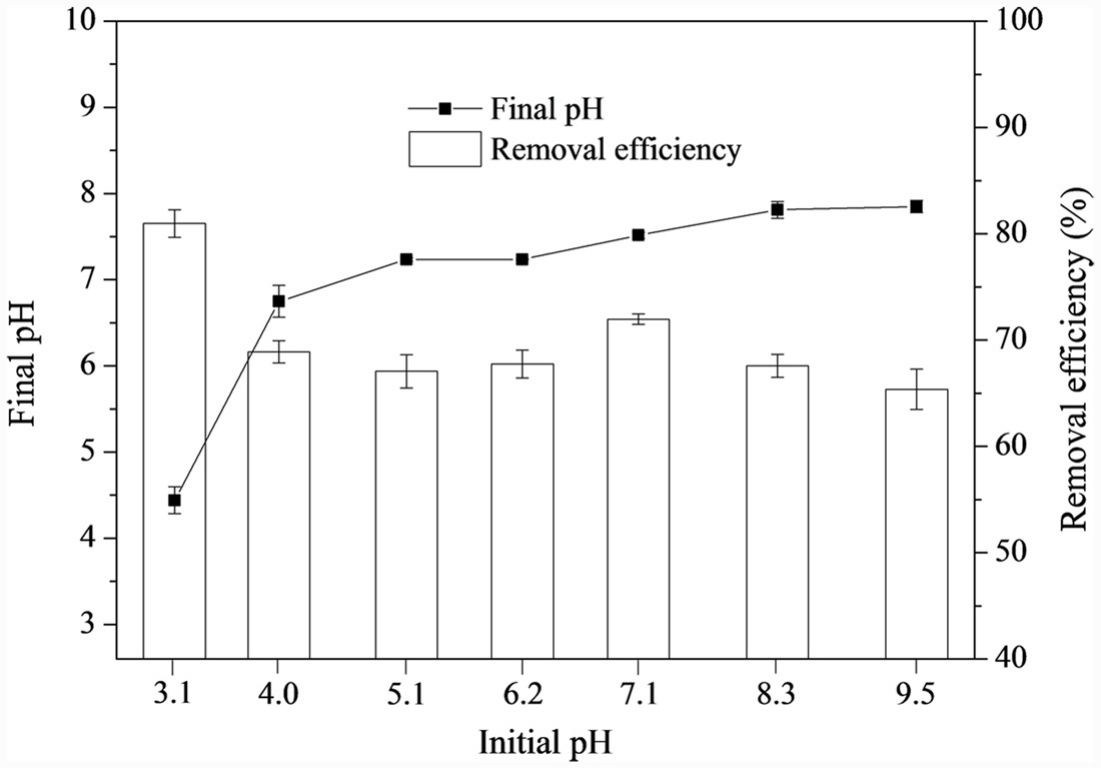
Effect of initial pH on phosphorus removal by LH pine needles.

The pH value is important in adsorption, because it affects the species of phosphorus ions and the ionization of active functional groups on sorbent surfaces [45]. As Fig 7 shows, at the initial phsophorus concentration of 10 mg/L, more than 65% of the phosphorus was removed at a pH range of 3 to 9.5, suggesting that the application of LH pine needles as an adsorbent could be performed in a wide pH range; the hightest removal efficiency was 81% at pH 3.1.

Phosphorus can exist in different ionic species, including H_2_PO_4_^-^, HPO_4_^2-^ and PO_4_^3-^ ions, depending on the pH of the bulk solution [34]. At pH 3.1, the dominant species were H_2_PO_4_^-^ with a small amount of H_3_PO_4_. Monovalent H_2_PO_4_^-^ may be strongly adsorbed onto LH pine needles by the intensive electrostatic interaction, or exchanged with hydroxyl groups arising from ionized Lanthanum hydroxide (La(OH)_2_^+^) deposited on the LH pine needles working as active sites. This could release OH^-^ to the solution, leading to the increased pH value [32]. Meanwhile, H_2_PO_4_^-^ could be protonated to H_3_PO_4_, which may replace the neutral water molecule from the hydrated lanthanum ions loaded onto the LH pine needles [16].

This could explain why the removal efficiency was higher in the lower pH range. When the pH value increased to 9.5, the removal efficiency did not dramatically decrease; this is not consistent with other adsorption research using La-modified adsorbents [24, 26, 32]. The OH^-^ brought about competitive adsorption, blocking the adsorption process and decreasing the removal efficiency. Meanwhile, lanthanum hydroxide protonation became weaker, and the lanthanum was in the insoluble form of La(OH)_3_ when the pH value was higher than 7.6. This state was unfavorable for adsorption and precipitation [25]. However, the OH^-^ ion could complex with functional groups, such as cellulose, to form cellulose-OH^-^ links [46]. This would reduce OH^-^ concentrations and ligand exchange to promote adsorption and keep the final pH increasing slowly from 6.8 to 7.9, while the initial solution pH increased from 4.0 to 9.5. In turn, this process could keep removal efficiency over 65% in the alkaline region.

### 3.7 Effect of competing anions

Testing the adsorption selectivity of a new adsorbent is vital before applying in practical use. Here, SO_4_^2-^, NO_3_^-^, Cl^-^, CO_3_^2-^ were chosen as interfering anions to test LH pine needle selectivity; As shown in Table 5, phosphorus removal efficiencies increase with the increasing concentrations of SO_4_^2-^, NO_3_^-^, Cl^-^ and achieve complete removal when the interfering anions are at concentrations of 100 mg/L. For CO_3_^2-^, the removal efficiency clearly decreases as the CO_3_^2-^ concentration increases. This may be because the K_sp_ of La_2_(CO_3_)_3_ (3.98×10^−34^) is lower than that of LaPO_4_ (3.7×10^−23^) forming La_2_(CO_3_)_3_ sediment more easily, and decreasing phosphorus removal efficiency [32].

**Table 5:** Effect of competing anions on the phosphorus removal efficiency (%) of LH pine needles.

	Competing anion concentration (mg/L)					
	0	1	5	10	50	100
CO_3_^2-^	90.2	89.5	89.1	87.6	85.8	73.4
SO_4_^2-^	90.2	91.7	89.3	93.9	98.2	100
NO_3_^-^	90.2	86.9	90.5	91.5	97.5	100
Cl^-^	90.2	91.8	90.1	94.2	100	100

### 3.8 Release of La and organic material

The ICP analysis was used to determine the concentration of the loosely bound La in the treated solution. As expected, the concentration of residual lanthanum ions was very low. The concentration of residual lanthanum ions was 1.61, 0.32 and 0.05 mg/L when the initial concentration of phosphate solution was 5, 20 and 50 mg P/L, respectively, and the corresponding concentration of lanthanum was thought nontoxic and environment-friendly by previous studies [47, 48]. This indicated most of the lanthanum ions were bounded on pine needle. Considering the low concentration of residual lanthanum ions, LPN could be considered an environment-friendly material for the removal of phosphate.

**Fig 8:**
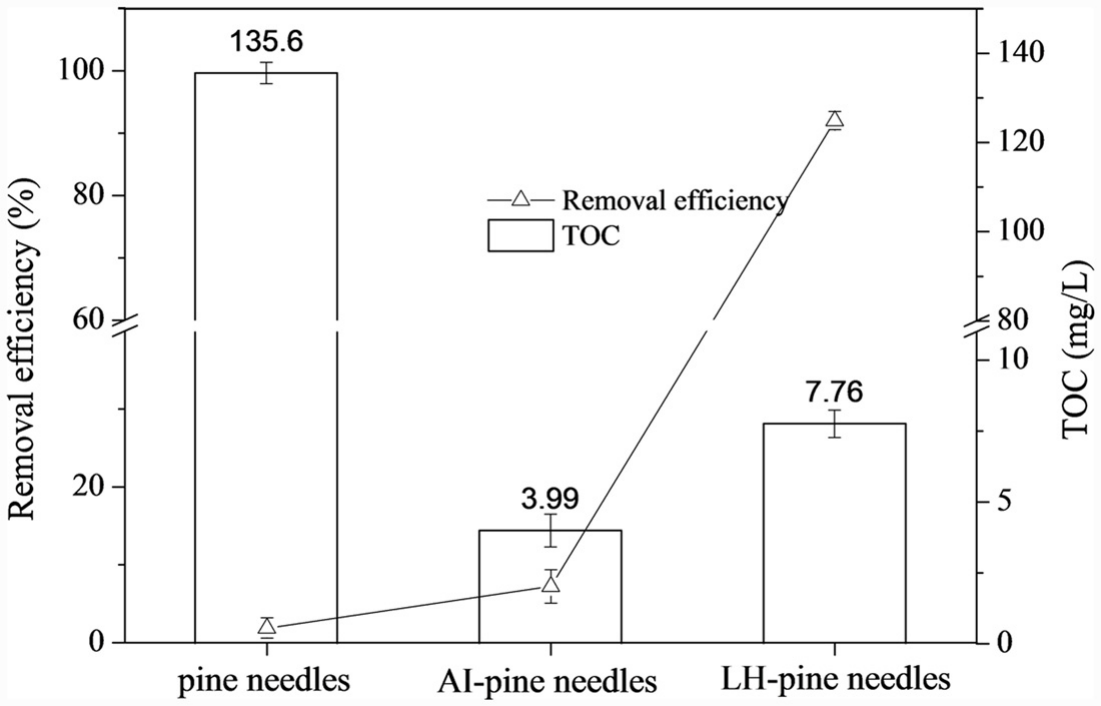
Comparison of total organic carbon (TOC) values and phosphorus removal efficiency of pine needles, AI pine needles and LH pine needles.

In addition, a TOC analysis determined the organic material release in the phosphorus removal process. As Fig 8 shows, AI pretreatment and La-modification significantly reduced the release of organic compounds. The TOC value of the LH pine needles was 7.8 mg/L; the TOC value of the AI-pine needles was 4.0 mg/L. The crushed pine needles without any treatment had the maximum TOC, of approximately 136 mg/L, which is approximately 17 times that of LH pine needles. The differences of TOC in the crushed and themodified pine needles (AI and LH), suggest that the AI pretreatment decomposed the pine needle surfaces and extracted the most organic compounds.

## Conclusions

In this study, AI pine needles showed a poor ability to adsorb low concentrations of phosphorus; LH pine needles showed higher removal efficiency. SEM photographs and EDAX analysis clearly showed that chemical treatment and LH precipitation resulted in many pores and adsorption site production. Sorption kinetics followed a pseudo first-order model and intra-particle diffusion model; the endothermic adsorption process could be divided into three phases. The phosphorus adsorption on LH pine needles showed a successful fit agaist Langmuir models. The maximum phosphorus capacity for LH pine needles was approximately 4.8 mg P/g at 298 K. LH pine needles demonstrated effective removal abilities across a wide pH range, with particularly strong abilities in the low pH range. There was only a slight inhibition of nitrate, sulfate and chloride, with the exception of carbonate in the co-existing anions competition tests. After chemical treatment, concentrations of released soluble organic compounds were significantly reduced.

## Supporting Information

S1 Fig EDAX spectrum of LH pine needle. (DOC)
