# Supplementary figures and images for: Removing Phosphorus from Aqueous Solutions Using Lanthanum Modified Pine Needles
Source: PLoS One. 2015 Dec 2;10(12):e0142700. doi: 10.1371/journal.pone.0142700 (PMC4668053; doi:10.1371/journal.pone.0142700)

**Supporting information**


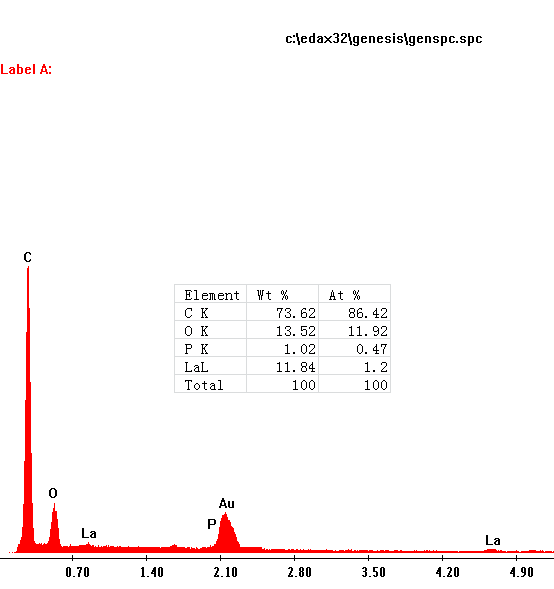


S1 Fig EDAX spectrum of LPN

Supplement: S1 Fig — (DOC) [file pone.0142700.s001.doc]
